# A Review on Microbial Products and Their Perspective Application as Antimicrobial Agents

**DOI:** 10.3390/biom11121860

**Published:** 2021-12-10

**Authors:** Alka Rani, Khem Chand Saini, Felix Bast, Sunita Varjani, Sanjeet Mehariya, Shashi Kant Bhatia, Neeta Sharma, Christiane Funk

**Affiliations:** 1 Department of Botany, School of Basic and Applied Sciences, Central University of Punjab, Bathinda 151401, India; alkaraniraj@gmail.com (A.R.); ksaini523@gmail.com (K.C.S.); 2 Gujarat Pollution Control Board, Gandhinagar 382010, India; drsvs18@gmail.com; 3 Department of Chemistry, Umeå University, 90187 Umeå, Sweden; christiane.funk@umu.se; 4 Department of Biological Engineering, College of Engineering, Konkuk University, Seoul 05029, Korea; 5 ENEA, Italian National Agency for New Technologies, Energy and Sustainable Economic Development, Department of Sustainability-CR Trisaia, SS Jonica 106, km 419 + 500, 75026 Rotondella, Italy; neeta.sharma@enea.it

**Keywords:** bacteriocins, lipopeptides, halocin, chlorellin, filamentous fungi, microalgae

## Abstract

Microorganisms including actinomycetes, archaea, bacteria, fungi, yeast, and microalgae are an auspicious source of vital bioactive compounds. In this review, the existing research regarding antimicrobial molecules from microorganisms is summarized. The potential antimicrobial compounds from actinomycetes, particularly *Streptomyces* spp.; archaea; fungi including endophytic, filamentous, and marine-derived fungi, mushroom; and microalgae are briefly described. Furthermore, this review briefly summarizes bacteriocins, halocins, sulfolobicin, etc., that target multiple-drug resistant pathogens and considers next-generation antibiotics. This review highlights the possibility of using microorganisms as an antimicrobial resource for biotechnological, nutraceutical, and pharmaceutical applications. However, more investigations are required to isolate, separate, purify, and characterize these bioactive compounds and transfer these primary drugs into clinically approved antibiotics.

## 1. Introduction

For the last few decades, antibiotics have saved millions of lives, but the prevalence of multidrug resistance (MDR) in microbial strains, nullifying the effects of antibiotics is an expected consequence of antibiotic abuse. The emergence and prevalence of antibiotic-resistant microbial strains remain one of the major health issues of the 21st century, creating pressure on natural microbiota. The ESKAPE pathogens (*Enterococcus faecium*, *Staphylococcus aureus*, *Klebsiella pneumoniae*, *Acinetobacter baumannii*, *Pseudomonas aeruginosa*, and *Enterobacter* species) are one of the greatest challenges faced by medical practices as many of them are multidrug-resistant isolates [1]. The US Centres for Disease Control and Prevention (CDC) classified the most concerning antimicrobial resistance (AMR) threats, cataloguing carbapenem-resistant *P. aeruginosa, Clostridium difficile*, and *A. baumannii*; MDR *Neisseria gonorrhoeae* and carbapenem- and cephalosporin-resistant *Enterobacteriaceae* as “urgent” threats [2], requiring urgent measures to deal with the situation. Pendleton et al. [3] provide a contemporary summary and clinically relevant information on the ESKAPE pathogens. In contrast, a detailed description regarding the antimicrobial resistance mechanisms of ESKAPE pathogens was illustrated by Santajit and Indrawattana [1], and can be used as a tool and applied to emerging MDR pathogens. Mulani et al. [4] highlight the use of therapies, including the combination of antibiotics, bacteriophages, antimicrobial peptides, nanomedicines, and photodynamic light therapy to overcome the limitations of individual therapy. These advanced and combinatorial therapies could be used as an alternate solution to combat AMR. 

**Figure 1:**
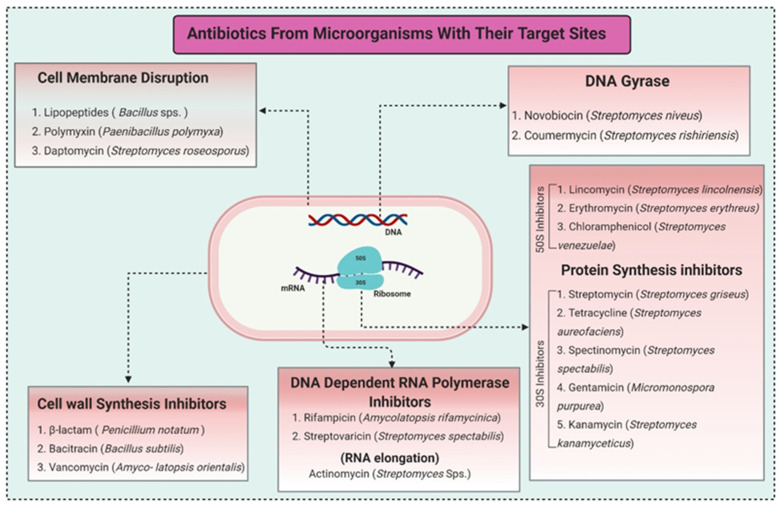
Antibiotics reported from different microorganisms with their target sites. Adapted from: [11].

Due to the increased consumption of livestock products in middle-income countries, antimicrobial consumption will increase up to 67%, and up to two-fold in India, Brazil, China, Russia, and South Africa, by 2030 [5]. The current review recapitulates the microbial metabolites, counting growth hormones, pigments, antibiotics, etc., that have become significant sources for life-saving drugs. Most microbial metabolites hold specific antimicrobial potential and act at particular target sites (Figure 1); thereby, they can be an attentive source for biotechnological applications, specifically for pharmaceuticals and nutraceuticals [6]. During the late 1980s, a shift from chemical synthesis in drug discovery from nature to the laboratory bench occurred, resulting in the discovery of approximately 50% natural drugs from 1981 to 2010 [7]. One of them was prodigiosin, an antimicrobial pigment produced by the marine bacterium *Vibrio ruber*, which induces autolytic activity in the *Bacillus subtilis.* Similarly, lantibiotics from Gram-positive bacteria were bioengineered to increase their effectiveness against a wide range of bacterial strains and to improve their stability while transmitting through the gastrointestinal (GI) tract making them protease-resistant [8].

Biofilms formed by bacteria are ubiquitous and are a part of their survival mechanisms. Biofilms have been involved in many clinical infections, such as atherosclerosis, pharyngitis, laryngitis, pertussis, bacterial vaginosis, etc. [9]. Although many bacterial strains are responsible for causing infections and diseases, bacterial antimicrobial compounds are reported as antifungal, antiviral, etc. We briefly highlighted the bacteriocins from lactic acid bacteria (LAB) which can disrupt the cell membrane integrity or inhibit cell wall synthesis, protein, and nucleic acid synthesis in pathogens. A recent study by Ting et al. [10] updated the epidemiology of the infectious keratitis (IK), the leading cause of corneal blindness; its causative microorganisms including bacteria, virus, fungi, parasites, and polymicrobial infections are major risk factors associated with the treatment of IK. Antimicrobial compounds such as vinaceuline, bafilomycin, antimycin, and other anti-methicillin-resistant *S. aureus* (MRSA) compounds synthesized by *Streptomyces* spp., which act antagonistically against different microbial strains are discussed in the present review. The current review also focuses on the halocins and sulfolobicin reported from archaea in a later section. Furthermore, the antimicrobials reported from endophytic, filamentous and marine-derived fungi along with mushrooms and microalgae are summarized. Microalgae act as a potential source of antimicrobial substances due to the synthesis of indoles, acetogenins, terpenes, phenols, and volatile halogenated hydrocarbons, which are also discussed. Hence, this review documents the potential antimicrobial compounds discovered from all possible microbial resources, including microorganisms inhabiting extreme habitats.

## 2. Bacteria

Bacterial antimicrobial compounds have been used traditionally for numerous reasons, including delaying the spoilage of food material or crops by plant pathogens in agriculture, and extending the shelf life of products in the food industry [12]. Compared to terrestrial bacteria, marine bacteria have many unique secondary metabolites due to their more complex and biologically competitive environment and their unique pressure, temperature, salinity, oxygen, light, and pH conditions. These factors make them a rich source of effective antibiotics. 

Many researchers have isolated and identified various antimicrobial compounds from marine bacteria against drug-resistant pathogens [13]. *Marinomonas mediterranea*, a marine bacterium isolated from the Mediterranean Sea at the Murcia coast has antagonistic activity against *Pseudomonas* sp. and *S. aureus* resistance to ceftazidime and meticillin antibiotics, respectively [14]. Ayuningrum et al. [15] isolated isatin from the marine bacterium *Pseudoalteromonas rubra* TKJD 22 associated with a marine tunicate, which has antibacterial activity against MDR pathogens including MDR *E. coli*, *B. cereus, Micrococcus luteus*, and *B. megaterium*. Ieodoglucomide and ieodoglycolipid isolated from the ethyl acetate extract of a marine-derived *Bacillus licheniformis* bears antifungal activity against the plant pathogens *Colletotrichum acutatum* and *Botrytis cinerea,* along with the human pathogen *Candid albicans* [16]. Similarly, janthinopolyenemycin A and B polyketides were isolated from the proteobacterium *Janthinobacterium* spp., strains ZZ145 and ZZ148, respectively, by Anjum et al. [17], who found that they hinder the growth of *C. albicans*. Schulze et al. [18] utilized a genome-assisted discovery strategy to isolate three macrolactams, lobosamides A, B, and C from *Micromonospora* sp. RL09-050-HVF-A. Among them, lobosamides A and B have antagonist activity against the microbial agent of African trypanosomiasis i.e., *Trypanosoma brucei*, whereas, lobosamide C has no bioactivity. Zhang et al. [19] reported Streptoseomycin, a macrolactone from the *Streptomyces seoulensis* A01, having specific activity against microaerophilic bacteria *Helicobacter pylori*. Bacicyclin, a cyclic peptide, was isolated from a *Bacillus* sp. strain BC028 associated with mussel (*Mytilus edulis*), it was found that it inhibits the growth of *Enterococcus faecalis* and *S. aureus* with minimal inhibitory concentration (MIC) values of 8 and 12 µM, respectively. In addition, it is used to design analogs with increased antibiotic efficacy [20].

*Bacillus* strains from both marine and terrestrial environments are widely known to produce extensive biocontrol metabolites, which include the ribosomally synthesized antimicrobial peptides (bacteriocins) [21], as well as non-ribosomally synthesized peptides (NRPs) and polyketides (PKs) [22]. 

### 2.1. Ribosomally Synthesized Antimicrobial Peptides (Bacteriocins) and Bacteriocin-Like Inhibitory Substances (BLIS)

**Figure 2:**
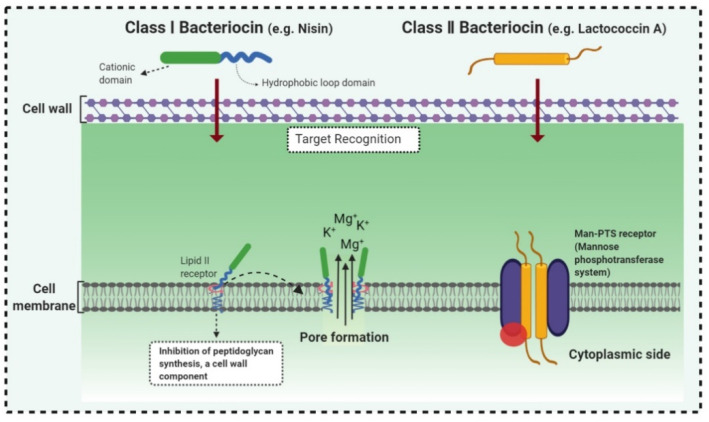
Mode of action of bacteriocins. Inhibition of cell wall synthesis: class II bacteriocins (e.g., lactococcin) cross the cell wall and bind with the pore-forming receptor in the mannose-phosphotransferase (man-PTS), resulting in the pore formation in the cell membrane. Pore formation: class I bacteriocins, (e.g., nisin) can follow both mechanisms. Nisin generated pores in the cell membrane resulting in the efflux of ions (K^+^ and Mg^2+^), amino acids (glutamic acid, lysin), generating proton motive force dissipation and ultimately causes cell death. Adapted from: [50,51].

Bacteriocins are antimicrobial ribosomal peptides reported from all major lineages of bacteria and some members of archaea. Gram-negative intestinal bacteria *Escherichia coli* produces bacteriocidal proteins, colicins, larger than 20 kDa which are antagonistic against zoonotic strains and might establish a defence line against multidrug-resistant strains. [23]. Bacteriocins have attracted increasing attention because of their use as a food preservative and therapeutic antibiotic. Furthermore, they have also received attention because they have a rapid-acting mechanism by forming pores in the membrane of target bacterial cells, even at very low concentrations (Figure 2). The recently reported bacteriocins along with their characteristics are presented in Table 1. 

Hoyt et al. [24] isolated the first marine bacteriocin from *Vibrio harveyi* after screening 795 strains of *Vibrio* sp. from Galveston Island (Texas, USA). This laid the foundation for multiple studies focused on the identification and biochemical characterization of new bacteriocins and bacteriocin-like compounds. Genera of marine bacteria producing bacteriocins include *Aeromonas, Bacillus, Burkholderia, Lactococcus, Pseudomonas, Photobacterium, Pediococcus, Enterococcus, Stenotrophomonas, Carnobacterium, Pseudoalteromonas, Streptomyces,* etc. The major difference between marine and terrestrial bacteriocins is that marine bacteriocins are resistant to high and low temperatures, osmotic stress, and various proteolytic enzymes and organic solvents, whereas terrestrial bacteriocins are not [25].

Bacteriocins from lactic acid bacteria (LAB) have gained significant attention due to their food-grade quality and industrial significance. LAB are live microorganisms which when administered in acceptable quantities in a host, trigger a health benefit by promoting and sustaining a strong immune system. Thus, LAB benefits consumers nutritionally and acts as an immunity booster against diseases and infections. LAB and its by-products are generally regarded as safe (GRAS) as a human food component by the U.S. Food and Drug Administration (FDA). Hence it is safer to use LAB bacteriocin to constrain the growth of pathogenic/undesirable bacteria [26]. Lozo et al. [27] isolated the bacteria *Lactobacillus paracasei* from customarily homemade white-pickled cheese and reported that it produces bacteriocin 217 (Bac217), exhibiting antimicrobial activity against *P. aeruginosa, Bacillus cereus, Salmonella* sp., and *S. aureus*. 

A study by Drissi et al. [28] suggests that bacteriocins are widespread across the human GI tract, with 317 microbial genomes encoding maximum bacteriocins of class I (44%) as compared to classes II (38.6%) and III (17.3%). Furthermore, they elaborated the bacteriocins produced by gut microbiota, i.e., class I bacteriocins display low antimicrobial activity, whereas maximum class II bacteriocins were reported from bacteria not occurring in the gut. Similarly, Leite et al. [29] described BLIS produced by *B. cereus* LFB-FIOCRUZ 1640 with activity against *Listeria monocytoges* and other *Bacillus* sp. in pineapple pulp and found that it can be used as a potential food bio preservative. Recently, Pircalabioru et al. [30] comprehensively reviewed bacteriocins’ potential as an antimicrobial agent against infections mainly due to resistant pathogens i.e., MRSA. In contrast, Jawan et al. [31] suggest that BLIS from *L. lactis* Gh1 inhibits the growth of *L. monocytogenes* and can be used in the food industry as functional foods for the preparation of starter culture and probiotic products. In addition, BLIS from *B. subtilis* BSC35 inhibits *Clostridium perfringens*; therefore, it can be used to control *C. perfringens* in fermented foods [32].

**Table 1:** List of recently reported Bacteriocins.

Type	Characteristics		Example	Producer	Mode of Action	References
Bacteriocin type I	Lantibiotics, very small (<5 kDa) peptides containing lanthionine and β-methyllanthionine		Nisin Z and Q, Enterocin W Nukacin ISK-1	*Lactococcus lactis*	Membrane permeabilization forming pore	[33]
Bacteriocin type II	Small (<10 kDa), non-lanthionine-containing peptides					
	IIa	heat-stable peptides synthesized as a precursor and processed after two glycine residues, antilisterial, bear consensus sequence YGNGV-C at the N-terminal	Enterocin NKR-5-3C, Enterocin A, Leucocin A, Munditicin	*Pediococcuspentosaceus,* *P. Acidilactici* and *L. sakei*	Membrane permeabilization forming pore	[34]
	IIb	Two-component systems: two different peptides work together and generate an active poration complex	Lactococcin Q, Enterocin NKR-5-3AZ, Enterocin X	*L. lactis sub* sp. *cremoris, L. plantarum*	Membrane permeabilization forming pore	[30,35]
	IIc	N- and C- termini are covalently linked, generating a circular bacteriocin	Lactocyclicin Q, Leucocyclicin Q	*L. gasseri,* *Enterococcus* *faecalis, L. garvieae*	Membrane permeabilization forming pore	[36]
	IId	Other class II bacteriocins, including unmodified, *sec*-dependent bacteriocins and leaderless, non-pediocin-like bacteriocins	Lacticin Q and Z, Weissellicin Y and M, Leucocin Q and N, Bactofencin A, LsbB	*L. salivarius, L. lactis* *Sub* sp. *Lactis*		[30,37]
Bacteriocin type III	Large peptides, sensitive to heat			*L. crispatus, L. helveticus,* *E. faecalis*		[38]
	IIIa	27 kDa, heat-labile protein	Lysostaphin and enterolysin A	*S. simulans* biovar Staphylolyticus, *Enterococcus faecalis*	Cell-wall degradation	[39]
	IIIb		Helveticin J	* Lactobacillus helveticus *	Disrupt membrane potential, which causes ATP efflux	[40]

Unfortunately, many factors cause a reduction in BLIS antimicrobial activity affecting the efficacy of bacteriocins. Such factors include the advent of bacteriocin-resistant strains, conditions that are destabilizing its biological activities such as oxidation processes, poor solubility, proteases or inactivation by other additives, and pH or temperature. Therefore, it is necessary to develop an antimicrobial system that minimizes these drawbacks and maximizes bacteriocins’ bioprotective potential.

Nisin belongs to type I bacteriocin and is the first antimicrobial peptide from *Lactococcus* and *Streptococcus* sp., it has been regarded as GRAS by both the FDA and the WHO [41]. Nisin has been used to inhibit microbial growth in beef, ground beef, sausages, liquid whole eggs, and poultry. It was reported that when nisin was crosslinked to chitosan, minimum inhibitory concentration (MIC) decreased from 48 μg/mL to 40 μg/mL for *Staphylococcus aureus* ATCC6538. The antimicrobial activity of nisin increased after crosslinking with a lesser concentration of chitosan i.e., the ratio of 200:1, thereby allowing better penetration into the lipid membrane [42]. The antibacterial constancy of nisin was successfully enhanced after its conjugation with gellan. Therefore, this conjugate can be an encouraging biomaterial for wound dressings and transplant coatings [43]. A study revealed the proficiency of nisin in combination with polymyxin in combating *P. aeruginosa* biofilms and reducing the dose of polymyxin required to interrupt *P. aeruginosa* biofilms [44]. Polymyxin might facilitate the transfer of nisin to its target. Along with nisin’s synergistic action with polymyxin and clarithromycin against *P. aeruginosa* and other non–*β*-lactam antibiotics against MRSA [45] and strains of vancomycin-resistant enterococci [46] were also reported. Webber et al. [47] embedded 0.89 µg cm^−2^ of positively charged nisin Z within polyelectrolyte multilayers (PEMs) i.e., nine layers of carrageenan (CAR) and chitosan (CS), forming a 4.5 bilayer film with antimicrobial activity against *S. aureus* and MRSA. Therefore, the antimicrobial potential of CAR/CS multilayers helps to realize its applicability within food, pharmaceutical, and biomedical industries [47]. Although nisin has a broad range of biomedical applications and is used in food bio preservation, further justification of nisin’s practicality and evaluation of its efficacy in biomedical fields will require in vivo and in vitro studies.

The mechanism of action of bacteriocins depends on the bacteriocin receptor molecules used. All bacteriocins from the same class do not follow a similar mode of action. Bacteriocins might utilize the same receptors but their mode of interaction with these receptor molecules and the aftereffect on the target cell could be quite different. For example, as described by Kjos et al. [48], bacteriocins lactococcin A and lactococcin Z share 55.6% similarity in the N-terminal region, both use IIC and IID components of mannose phosphotransferase system (man-PTS) as receptor molecules on target cells. As depicted in Figure 2, lactococcin A results in pore formation and dissipates the cell membrane potential, whereas lactococcin Z kills target cells without following any of these mechanisms. Lozo et al. [49] comprehensively represented the bacteriocins classification-based receptor molecules in target cells according to structural similarity within the same receptor molecule.

Class I bacteriocins are cationic lantibiotics (e.g., nisin) that electrostatically bind with the negatively charged membrane phospholipids II, allowing further interaction of bacteriocin′s hydrophobic domain with the target cytoplasmic membrane (lipid II), thereby preventing the biosynthesis of peptidoglycan [30,52]. Similarly, class III bacteriocins, enterolysin A with N-terminal endopeptidase domain and a C-terminal substrate recognition domain, exhibit antimicrobial activity against streptococci by cleaving the peptidoglycan cross-links between l-alanine and d-glutamic acid of the stem peptide and between l-lysine of the stem peptide and d-aspartic acid of the interpeptide bridge of the target cell [53].

### 2.2. Non-Ribosomal Synthesized Peptides (NRPs) and Polyketides (PKs)

NRPs and PKs include a range of cyclic, linear, and branched compounds, synthesized by composite enzymes viz. non-ribosomal peptide synthetases (NRPS), polyketide synthetases (PKS), and hybrid of NRPS/PKS, respectively [22,54]. Lipopeptides (LPs) are NRPs produced by Bacillales; LPs have significant antimicrobial activity [55]. LAB is considered the primary producer of ribosomally synthesized antimicrobial peptides, as reviewed by Alvarez-Sieiro et al. [53] and Pircalabioru et al. [30]. However, the classification scheme for antimicrobial compounds produced by *Bacillus* is not explored in comparison to LAB. Caulier et al. [22] reviewed and updated the antimicrobial metabolites classification from the *B. subtilis* group based on biosynthetic pathway and chemical nature. Zhao et al. [21] acknowledged 31 types of PKs, NRPs, and NRPS/PKS hybrid synthesized antimicrobials using antiSMASH.

### 2.3. Lipopeptides (LPs)

LPs occur naturally and are of bacterial origin, contain a hydrophobic long alkyl chain that associates with a hydrophilic polypeptide, and they form a cyclic or linear structure. Traditional LPs including the iturins, surfactins, and fengycins (Table 2) produced from *Bacillus* species are homologs that differ in length, branching pattern, and saturation of their acyl chain. LPs comprise anionic (e.g., surfactin and daptomycin) or cationic (e.g., colistin and polymixin B) peptide motif, dictating the range of their activity. As demonstrated by Perez et al. [56] *Bacillus* sp. P5 synthesize LPs iturin A, bacteriocin subtilosin A, and surfactin exhibiting antimicrobial activity against *L. monocytogenes* and *B. cereus*, along with the antifungal activity. A study by Kourmentza et al. [57] reported that a mixture of mycosubtilin and mycosubtilin/surfactin LPs inhibit the growth of filamentous fungi *Byssochlamys fulva* and *Paecilomyces variotti*, with MICs of 1–16 mg/L and *Candida krusei* with MIC of 16–64 mg/L.

**Table 2:** Various types of LPs and their characteristics.

Type	Characteristic Features	Molecular Weight	Chemical Structure	Producer	Applicability	References
Surfactin	Cyclic heptapeptide is an antibiotic with seven amino acids i.e., Glu-Leu-Leu-Val-Asp-LeuLeu (ELLVDLL). A, B, and C types varying according to their amino acid sequences.	~1.03 kDa	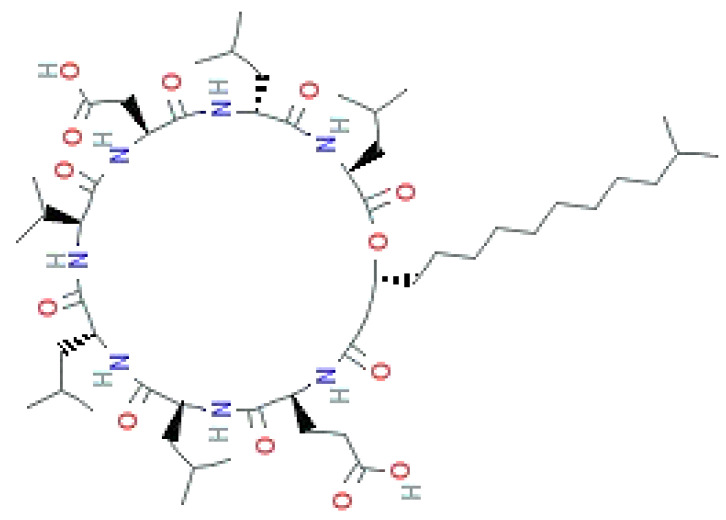	*B. subtilis* MSH1 and *B. amyloliquefaciens* ES-2	Antimicrobial, antifungal, insecticidal, antimycoplasma, hemolysis, and formation of ion channels in lipid membranes.	[58]
Iturin	Contains two major parts: a peptide part composed of 7 amino acid residues (Asn-Tyr-Asn-Gln-Pro-Asn-Ser) and 11-12 carbons hydrophobic tail. Example Iturin A, Bacillomycin D, Bacillomycin L, Mycosubtilin	~1.04 kDa	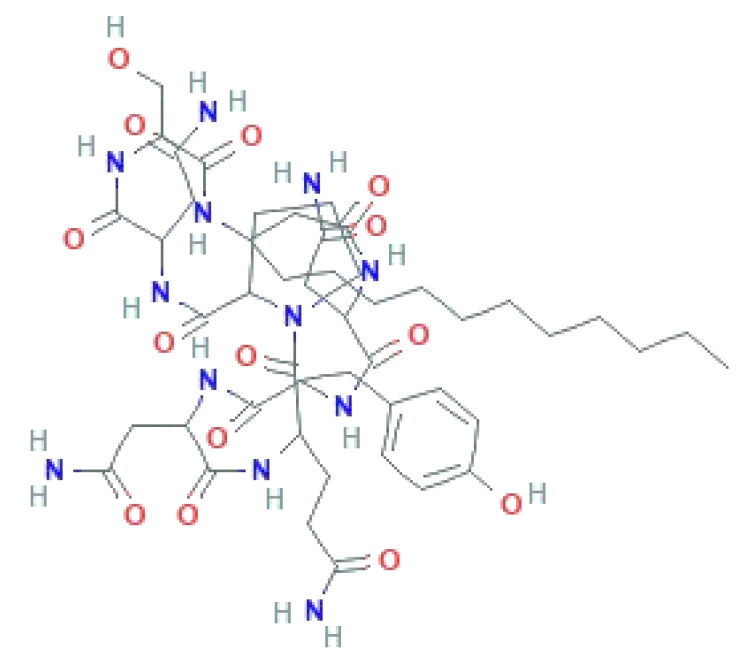	*B. subtilis*, *B. amyloliquefaciens* B128 and *B. amyloliquefaciens* BUZ-14	Antimicrobial and antifungal activities. Disrupt the membrane of yeast cells by increasing the electrical conductance of bimolecular lipid membranes.	[59]
Fengycin	An array of 10 amino acids with a lactone ring and a ß-hydroxy fatty acid linked to the N-terminus of a decapeptide. Example Plipastatin A and B	1463.7 g/mol	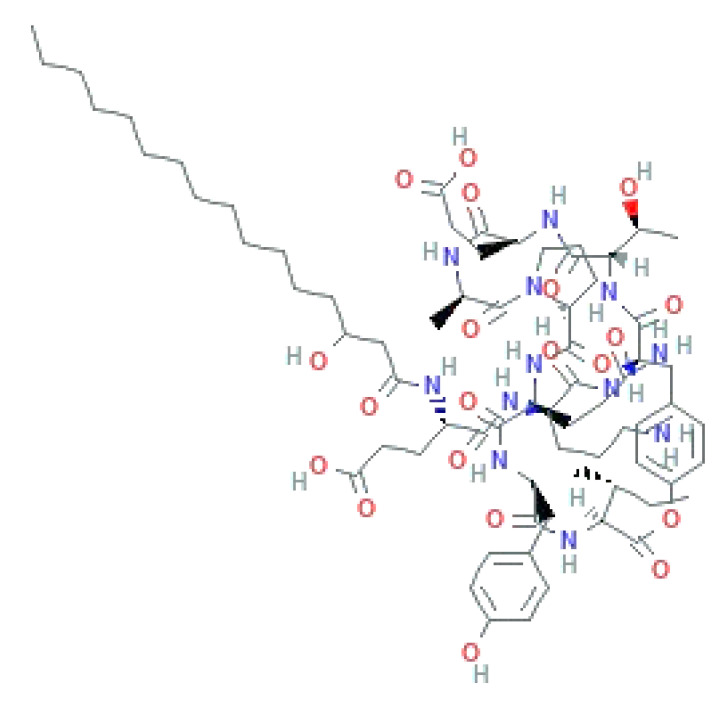	*B. subtilis*	Act as bioagents showing hypocholesterolemic activities, immuno-modulators; antibiotics, antiviral, and antitumor agents; toxins; and enzyme inhibitors	[60]

Surfactins, a cyclic heptapeptide that formulates a lactone bridge with *β*-hydroxy fatty acids, are the most potent biosurfactant. They display an array of activities including hemolytic, antiviral, anti-mycoplasma, and antibacterial [61]. Surfactin WH1 fungin from *Bacillus amyloliquefaciens* WH1 is an antifungal inhibiting glucan synthase that reduces the synthesis of callose on the fungal cell wall and binds to ATPase on the mitochondrial membrane, ultimately inducing apoptotic markers to stimulate the extracellular apoptotic pathway [62]. Many researchers claim that after inserting into the lipid bilayers, surfactin acts by forming voltage-independent channels in biofilms, distorting the membrane integrity and permeability of ions, i.e., K^+^ and Ca^2+^, causing membrane disruption [63].

Iturins are comprised of A, C, D, and E isoforms, bacillomycin D, F and L, and mycosubtilin that inhibit bacterial growth in the same manner as class I and class II bacteriocins [64]. A marine-derived *Bacillus velezensis* 11-5 produced a cyclic lipopeptide (CLP) iturin A, which is considered an antagonist against *Magnaporthe oryzae*, a rice pathogen [65]. Fengycin, an anti-fungal lipopeptide, isolated from *Bacillus* sp. *is* also called plipastatin. Both iturins and fengycins act as biocontrol agents preventing plant diseases and inhibiting the progression of a wide variety of plant fungal pathogens including *Aspergillus flavus*, *Rhizoctonia solani*, *Fusarium graminearum*, *Botritis cinerea,* and *Penicillium expansum* [66]. However, there is no doubt that LPs are a novel class of antibiotics exhibiting a wide range of activities. Therefore, detailed structural and functional knowledge is required to exploit them as potent antimicrobials, feed additives, and drug delivery systems.

## 3. Actinomycetes

Approximately 75% of the known industrial antibiotics and economically important compounds were obtained from the *Streptomyces* species [67]. Actinomycetes can synthesize antifungal, antiviral, antitumor, anti-inflammatory, antioxidants, immunosuppressive, plant-growth-promoting, and herbicidal compounds [68]. Among actinomycetes, *Streptomyces* is the most dominant because of a broad range of bioactive metabolites. Genus *Streptomyces* is classified into the family Streptomycetaceae based on its morphology and cell wall chemotype. *Streptomyces* spp. have filamentous hyphae, allowing them to efficiently utilize nutrients in the rhizosphere, enabling them to colonize and carry out a complex life cycle. *Streptomyces* spp. catabolizes complex molecules and substances, such as cellulose, lignocellulose, xylan, lignin, etc. to produce well-known bioactive compounds. The genus *Streptomyces* alone contributes approximately 7500 of the 10,000 known compounds from actinomycetes, whereas the other genera including *Actinomadura, Micromonospora, Nocardia, Saccharopolyspora, Actinoplanes* and *Streptosporangium* contribute approximately 2500 compounds [69]. Marine or terrestrial actinomycetes utilize enzymes polyketide synthases (PKS) or non-ribosomal peptide synthetases (NRPS) for the synthesis of metabolic bioactive compounds [70]. 

Pacios et al. [71] reviewed the importance of the *Streptomyces* genus as a prodigious producer of bioactive metabolites that act as a biological control against phytopathogenic bacteria. Widowati et al. [72] reported a new strain of marine actinomycetes, NPS12745 associated with marine sediment from the coast of San Diego, California, and after using 16S rRNA gene sequencing, NPS12745 was confirmed to be a fruitful strain of genus *Marinispora,* which produced ample new chlorinated bisindole pyrroles and their derivatives, including chromopyrrolic acid, which was earlier isolated from *Chromobacterium* *violaceum*. The first halogenated bisindole derivative was lynamicins A–E, having activity against several Gram-positive and Gram-negative bacteria, i.e., MSSA (methicillin-susceptible *Staphylococcus aureus*), MRSA, *S. epidermidis,* and *Enterococcus faecalis*, signifying a possible cure for nosocomial infections [73]. Siddharth and Vittal [69] isolated *Streptomyces* sp. S2A from the Gulf of Mannar, which have antagonistic activity against bacterial (*Micrococcus luteus, S. epidermidis, Klebsiella pneumoniae, Bacillus cereus,* and *S. aureus*) and fungal (*Fusarium moniliforme* and *Bipolaris maydis)* pathogens. A unique prenylated-indole derivative known as 3-acetonylidene-7-prenylindolin-2-one, hybrid isoprenoids, 3-cyanomethyl-6-prenylindole, 7-isoprenylindole-3-carboxylic acid, and 6-isoprenylindole-3-carboxylic acid were extracted from the *Streptomyces* sp. neau-D50. These antifungal compounds prevent the growth of phytopathogenic fungi *Corynespora cassiicola, Phytophthora capsica, Colletotrichum orbiculare,* and *Fusarium oxysporum* [74]. Djinni et al. [75] described *Streptomyces* *sundarbansensis* WR1L1S8, an endophyte sequestered from brown algae, yields an innovative anti-MRSA compound, [2-hydroxy-5-((6-hydroxy-4-oxo-4H-pyran-2-yl)methyl)-2-ropylchroman-4-one] beside three already reported polyketides, namely phaeochromycin B, C, and E, which are active against Gram-positive pathogenic MRSA. Sebak et al. [76] isolated *Streptomyces* sp. MS. 10 from Egyptian soil and reported the presence of saturated fatty acid through ^1^H NMR spectroscopy, which bears broad-spectrum antimicrobial activity against MRSA. Recently, Qureshi et al. [77] identified compounds, Actinomycin X_2_ and D, from *Streptomyces smyrnaeus* UKAQ_23 collected from the mangrove-sediment, with MIC of 1.56–12.5 µg/mL for non-MRSA and 3.125–12.5 µg/mL for MRSA, respectively. 

Yang et al. [78] isolate vinaceuline, a cyclopeptide, activity against bacteria, from the broth culture of endophytic *Streptomyces* sp. YIM64018 allied with *Paraboea sinensis*. The same team isolated a new benzamide, 2-amino-3, 4-dihydroxy-5-methoxybenzamide in 2015, from *Streptomyces* YIM67086 that attacks *E. coli* and *Candida albicans* (MICs of 64 and 32 μg/mL, respectively). Ding et al. [79] reported that 7, 3’-di-(c,c dimethylallyloxy)-5-hydroxy-40-methoxyflavone, an antifungal compound, from the broth culture of *Streptomyces* sp. MA-12 obstructs the growth of plant pathogens *Penicillium citrinum, Gibberella zeae*, and *Colletotrichum musae*. 

Lee et al. [80] isolated 87 actinomycetes species including *Streptomyces pluripotens* MUSC135T, that inhibit MRSA. This antibacterial metabolite-producing ability was confirmed by PKS (polyketide synthetase) and NRPS (non-ribosomal polyketide synthetase) gene detection process. *Streptomyces* sp. colonizing on root tissues produce ample antifungal and antibacterial compounds i.e., antimycin A18, phaeochromycin B, C and E, diastaphenazine, 3-acetonylidene-7-prenylindolin-2-one, and staurosporine, some of which are represented in Table 3. Similarly, Jaroszewicz et al. [81] isolated *Streptomyces* sp. M4_24 and M5_8 strains and identified the presence of dichloranthrabenzoxocinone and 4,10- or 10,12-dichloro-3-O-methylanthrabenzoxocinone, which are putative antimicrobial compounds. A newly discovered lipopeptide NRPS/PKS-derived colibrimycins, from *Streptomyces* sp. CS147, isolated from *Attini* ant niche displayed antagonism against virus protease [82]. An endophytic actinomycetes, VITGV01, isolated from a farm tomato plant produced different antibiotics on different media which were active against Gram-positive and Gram-negative bacteria including *B. subtilis, S. aureus*, *E. coli*, and *Klebsiella pneumoniae* [83]. The unique properties of rhizospheric actinomycetes which allow them to produce a diverse range of bioactive metabolites with antagonistic outcomes toward pathogens have led them to be a potent agent ensuring plant health.

Cycloserin, an antibiotic produced by *Streptomyces orchidaceus*, blocks protein synthesis and is used to treat tuberculosis in conjunction with other drugs [84]. Robertsen and Musiol-Kroll [85] reviewed the actinomycetes-derived polyketide drugs, such as erythromycin A, tetracyclines, rifamycin, tylosin, monensin A, amphotericin B, etc. with antimicrobial activity, including the source of the compounds, their structure, the biosynthetic mechanisms, and mode of action. However, the increasing rate of MDR requires the rediscovery of compounds from potential producers. However, many organisms require special cultivation conditions, therefore, many strategies need to be developed in order to overcome such barriers. Hug et al. [86] described the strategies and innovative methods such as advanced cultivation methods, genomics, metabolomics, and metagenomics-based approaches used to explore the new reservoir of actinomycetes and improve the efficacy of antimicrobial compounds. Hence, it was concluded that *Streptomyces* spp. can be used as a promising candidate with the potential to be scaled up for industrial production, which could benefit both the agricultural and pharmaceutical industry.

**Table 3:** Bioactive compounds from endophytic actinomycetes.

Endophytic Actinomycetes	Host	Bioactive Compounds	Structure	Bioactivity	References
*Streptomyces* sp. YIM64018	*Paraboea sinensis*	Vinaceuline	-	Antibacterial activity	[87]
*Streptomyces* sp. neau-D50	*Glycine max*	3-acetonylidene-7-prenylindolin-2-one, 7-isoprenylindole-3-carboxylic acid	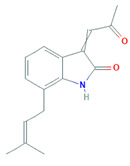	Cytotoxic and antifungal activities	[74]
*Streptomyces* sp. YIM56209	*Drymaria* *cordata*	Bafilomycin D, B1, B2, C1, C2, C1 amide and C2 amide	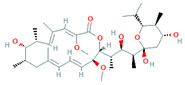	Antibacterial, antifungal, insecticidal, antihelmintic and cytotoxic activity	[88]
*Streptomyces diastaticus* Sub sp. *ardesiacus*	*Artemisia* *annua*	Diastaphenazines	-	Antibacterial and antifungal activity	[89]
*Streptomyces* sp. YIM67086	*Dysophylla stellata*	4-hydroxy-3-methoxybenzoic acid, p-hydroxytruxinic acid	-	Antifungal activity	[78]
*Microbispora* sp. LGMB259	*Vochysia* *divergens*	β-carboline or 1-vinyl-β-carboline-3-carboxylic acid	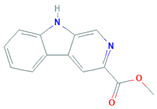	Antibacterial, antifungal and anticancer activity	[90]
*Streptomyces* sp. YIM66017	*Alpinia* *oxyphylla*	Yangjinhualine A and 2,6-dimethoxy terephthalic acid	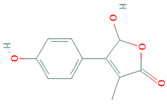	Radical scavenging activity	[91]
*Streptomyces albidoflavus* 07A-01824	*Bruguiera gymnorrhiza*	Antimycin A18	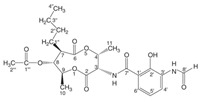	Antifungal activity	[92]
*Streptomyces* sp. VITMK1	mangrove soil	Pyrrolopyrazines	-	Antimicrobial	[93]
*Streptomyces* sp.		Diketopiperzines	-	Anti-H1N1 activity	[94]

## 4. Archaea

Archaeocins, is a proteinaceous antibiotic produced from archaea which mark the chronicled beginning in the series of antimicrobial compounds. The term “archaeocin” was used to differentiate the archaeal peptide and protein-based antibiotics from those produced by bacteria [95]. Only two phylogenetic groups have produced archaeocins (Table 4); one is euryarchaeal producing “halocins”, whereas the other group is crenarchaeal genus Sulfolobus producing “sulfolobicin” [96]. Valera et al. [97] reported halocins, the first proteinaceous antimicrobial compound from halophilic members of the archaeal domain. Archaeal protein VLL-28, from *Sulfolobus islandicus*, is the first archaeal antimicrobial peptide, possessing a broad-spectrum antibacterial and antifungal activity [98]. Until recently, very few reports were available on the characterization of antimicrobial compounds from archaea. Besse et al. [99] comprehensively reviewed the archaeocins and sulfolobicins antimicrobial peptides ribosomally-synthesized by archaea belonging to the order Halobacteriales and Sulfolobales, respectively. However, until recently halocin A4, G1, R1, H1 [100]; H2 [37]; H3, H5 [97]; H4 [101]; H6 [102]; C8 [103]; S8 [104]; HalR1 [105]; and Sech7a [106] have been considered up to their molecular level, however, their mode of action is not yet clearly understood [107]. Only some workers reported that halocins kill the indicator organisms by altering the cell permeability at membrane level followed by cell lysis. However, to date, only the mode of action mechanism of halocin H6/H7 produced by *Haloferax gibbonsii* was characterized. HalH6 specifically inhibits Na^+^/H^+^ antiporter and proton flux ultimately causing cell lysis and death [108]. 

H1 and H4 are proteinaceous halocins of roughly 30–40 kDa [109], whereas C8, H6, H7, R1, U1, and S8 are microhalocins which are smaller than 10kDa. Microhalocins are more vigorous than proteinaceous halocins since they are resistant to varying temperature, salinity, exposure to organic solvents, acids, and bases [109]. Halocins have wide-ranging activity against haloarchaea and members of the family Halobacteriaceae [110]. Mainly halocin production is prompted during the progression between exponential and stationary phases, with H1 being an exception, produced during the exponential phase of the growth cycle [111]. Recently, Sahli et al. [112] screened 81 halophilic strains collected from solar salterns of Algeria’s northern coast for the production of antimicrobial compounds, through partial 16S rRNA gene sequencing, these strains were recognized to belong to the *Haloferax* (*Hfx*) sp.

**Table 4:** Archaeocins reported from halobacteria.

Halocin	Producers	Size (kDa)	Origin	Active Against	Mode of Action	References
HalH1	*Haloferaxmediterranei* Xia3	31	Solar salterns, Alicante, Spain	Members of the Halobacteriales	Alter membrane permeability	[99]
HalH4	*Hfx. mediterranei* R4	34.9	Solar salterns, Tunisia	Members of the Halobacteriales, Strains of *Sulfolobus* sp.	Alter macromolecular synthesis, cell wall conformation, and Na^+^/H^+^ antiport inhibitor	[113]
HalH6	*Hfx. gibbonsii* Ma2.39	32	Solar salterns, Alicante, Spain	Members of the Halobacteriales	Alter intracellular osmotic balance, Na ^+^/H^+^ antiport inhibitor	[99]
HalS8	Haloarchaeal strain S8a, *Halobacterium salinarum* strain ETD5	3.58	Great Salt Lake, (Utah, United States)	*Halobacterium salinarum* NRC817, *Hbt*. sp. strain GRB and *Hfx. gibbonsii*	ND	[104,114]
HalC8	*Natrinema* sp. AS7092	7.4	Chaidan Salt Lake in Qinghai province, China		ND	[111,115]
HalR1	*Hbt. salinarum* GN101	3.8	Guerrero Negro, Mexico	Members of the Halobacteriales, Strains of *Sulfolobus* sp., *Methanosarcina thermophile*	ND	[37,99]
Sulfolobicins	*Sulfolobus* *Islandicus* HEN2/2	33.9 proprotein), 3.6 (mature)	Solfataric fields, Iceland	Strains of *Sulfolobus* sp.	ND	[116]

Note: ND: Note Detected or Not Reported.

Roscetto et al. [117] reported that VLL-28 damages the cell wall of *Candida albicans* and *C*. *parapsilosis* by binding to their cell surface. Kumar and Tiwari [118] purified halocin HA1 from *Haloferax larsenii* HA1 and HA3 from *H. larsenii* HA3; both were halocidal against *H. larsenii* HA10, instigating cellular distortion, releasing cell contents, and finally causing cell death. Because of these properties, it can be used for the preservation of leather hides and salted foods in the leather and food industries. Ghanmi et al. [114] isolated *Halobacterium salinarum* ETD5*, H. salinarum* ETD8, and *Haloterrigena thermotolerans* SS1R12 of the order Halobacteriales and reported that their antimicrobial activity is due to the production of a halocin, HalS8, a hydrophobic peptide. Quadri et al. [119] isolated archeal strain, *Natrinema gari,* the common producer of antimicrobial compounds, which after partial purification and characterization resembles the microhalocin HalC8. Besse et al. [115] confirmed that *Natrinema* sp. synthesizes Halocin C8, a 7.4 kDa peptide involving the genes *halC8*. 

Although many studies characterized the synthesis of halocins, the research concerning their structure and mode of action is still far behind in comparison to the antibiotics produced by other domains. Nowadays, when archaea gain more attention, it becomes necessary to explore their metabolites’, biosynthetic pathways, mode of action, etc., using the latest available technology. 

## 5. Fungi

In 1929, Alexander Fleming discovered the mold juice ‘Penicillin’ from *Penicillium notatum* fungus with an antibacterial activity [120]. Afterwards, several researchers started to search for a better strain to attain higher yields in easier growth conditions. After extensive research, *Penicillium chrysogenum* strains were considered for the commercial production of penicillin [121]. Revilla reported in 1986 the formation of the intermediate isopenicillin N in the course of penicillin G production in *P. chrysogenum* cultures [122], thereafter the formation of isopenicillin N/penicillin N and its late transformation to cephalosporin C in *Acremonium chrysogenum* [123]. Cephalosporins, a known antimicrobial agent, were purified from a marine fungus, *Cephalosporium acremonium* [124]. Recently, Li et al. [125] reported that pneumocandins, a lipohexapeptides of the echinocandin family, were produced by wild-type fungi *Glarea lozoyensis* and *Pezicula* (*Cryptosporiopsis*) species. Pneumocandins non-competitively bind to a catalytic unit of *β*-1,3-glucan synthase, resulting in osmotic uncertainty and cell lysis.

### 5.1. Endophytic Fungi

Huang et al. [126] discovered ten-membered lactones from endophytic fungus *Phomopsis* sp. YM 311483, with antifungal activity against *Aspergillus niger, Fusarium,* and *Botrytis cinere*. Endophytic *Fusarium* sp. from *Selaginella pollescens* collected from the Guanacaste conservation area of Costa Rica inhibit *C. albicans* [127]. The number of antimicrobial compounds were reported from the endophytic fungi, some of which are listed in Table 5.

### 5.2. Marine-Derived Fungi

In 2015 Meng et al. [133] discovered pyranonigrin F from fungus *Penicillium brocae* MA-231 allied with the *Avicennia marina,* a marine mangrove plant. Pyranonigrin F inhibits *S. aureus* (Gram-positive), *Vibrio harveyi,* and *Vibrio parahemolyticus* (Gram-negative bacteria), with considerably lower MIC values in comparison to the positive control (chloromycetin). Likewise, it is active against plant fungal pathogens *Alternaria brassicae* and *Colletotrichum gloeosprioides*, with improved MIC values compared to the positive control (bleomycin). Wu et al. [134] discovered Lindgomycin from *Lindgomyces* strains LF327 and KF970, reported from a sponge in the Baltic Sea, Germany, and Antarctica, respectively. Lindgomycin displayed antimicrobial activity against *S. aureus, S. epidermidis,* and methicillin-resistant *S. epidermidis* (MRSE). However, the inhibiting potential was two times less than the positive control chloramphenicol. It also constrains plant pathogenic bacterium *Xanthomonas campestris.* There is a never-ending list of antimicrobial compounds from marine fungi; a few of which are listed in Table 6, which displays their host, producer species, and bioactivity. 

Peniciadametizine A and Peniciadametizine B derivative of thiolated diketopiperazine was isolated from sponges-associated *Penicillium* sp. viz. *Penicillium adametzioides* AS-53 and *Penicillium* sp. LS54, respectively. Both derivatives inhibit *A. brassicae* (pathogenic fungus) with a MIC of 4.0 µg/mL and 32.0 μg/mL, respectively [138]. Communol A, G, and F extracted from *P. commune* 518 displayed antibacterial activities against *E. coli* with MIC values of 4.1, 23.8, and 6.4µM, respectively, and also against *E. aerogenes* [136]. Pyrrospirones were produced by marine-derived fungus *Penicillium* sp. ZZ380, isolated from *Pachygrapsus crassipes* which is a wild crab found on the seaside rocks of Putuo Mountain (Zhoushan, China). Pyrrospirones C-F, H, and I inhibit MRSA and *E. coli* having MIC values of 2.0–19.0 μg/mL [155]. Song et al. [156], following the previous lead, separated penicipyrrodiether A from a cultured marine fungal strain *Penicillium* sp. ZZ380 which inhibits *E. coli* and *S. aureus* with MIC of 34.0 and 5.0 μg/mL, respectively. These laboratory studies need to be directed toward developing the efficiency and effectiveness of isolated compounds that could benefit society in the long-term.

### 5.3. Mushrooms

Mushrooms are colonizing fungi belonging to division Eumycota and subdivision Basidiomycetes, characterized by the formation of basidiospores. Most of these macrofungi are edible, with culinary, nutritional, and medicinal characteristics, but many of them are not palatable or are poisonous [157]. Besides the nutritional and culinary properties, their antimicrobial activities attracted researchers seeking natural solutions to deal with the urgent requirements of food safety. Mushrooms have been publicly consumed for thousands of years due to their medicinal and nutritional properties. Secondary metabolites and extracts from mushrooms have recently attained considerable attention due to their anti-cancer, antioxidant, anti-inflammatory, antimicrobial, antidiabetic, and immunomodulatory properties. Approximately 1069 mushroom species have been consumed by people [158]. To date, numerous antimicrobial peptides have been acknowledged from mushrooms. Plectasin (endogenous peptide antibiotics), an antibacterial peptide, was extracted from *Pseudoplectania nigrella*. Mygind et al. [159] demonstrated the potent activity of recombinant plectasin against some Gram-positive *Streptococcus pneumoniae*. Wong et al. [160] described an antifungal peptide, cordymin isolated from medicinal mushroom *Cordyceps militaris,* which repressed mycelial growth of *Bipolaris maydis, Mycosphaerella arachidicola, Candida albicans,* and *Rhizoctonia solani* with IC_50_ values of 50 μM, 10 μM, 0.75 mM, and 80 μM, respectively. They also reported the remarkable pH stability (pH 6–13), thermostability (100 °C), and metal ion stability (10 mM Mg^2+^ and 10 mM Zn^2+^) of cordymin. An investigation by Gebreyohannes et al. [161] revealed that chloroform, ethanol, and hot water extract of *Auricularia* and *Termitomyces* sp. promisingly inhibited *E. coli*, *K. pneumoniae, C. parapsilosis*, and *S. aureus.* Poompouang and Suksomtip, [162] isolated an antifungal compound of 17 kDa from fruiting bodies of edible mushroom, *Lentinus squarrosulus,* inhibiting *Trichophyton mentagrophytes* and *T. rubrum,* a human fungal pathogen. More recently, Irshad et al. [163] comprehensively reviewed the synthesis and mode of action of polysaccharides silver nanoparticles (NPs) from *Pleurotus* mushroom. They characterized the NPs through ultraviolet-visible (UV–Vis), Fourier transformation infrared spectroscopy (FT-IR), scanning electron microscopy (SEM), energy dispersive spectroscopy (EDS), transmission electron microscopy (TEM), etc., and disclosed their promising antimicrobial efficiency. However, further studies are required in order to fortify and test these extracts and NPs against human and plant pathogenic microorganisms coupled with the purification and characterization of the compounds from mushrooms. 

Hamamoto et al. [164] screened the volatile compound, 3,4-dichloro-4-methoxy benzaldehyde (DCMB) from mycelia of *Porostereum spadiceum.* It remarkably inhibited the plant-pathogenic bacteria (*Clavibacter michiganensis* and *Ralstonia solanacearum*) and inhibited the conidial germination of plant-pathogenic fungi (*Alternaria brassicicola* and *Colletotrichum orbiculare*). However, further studies are essential to investigate its effects on plant-pathogens in vivo. Subrata et al. [165] reported that edible wild mushrooms’ methanolic extracts exhibited different levels of antimicrobial activities. A recent study by Sevindi [166] analysed the phenolic content of the wild edible mushroom *Melanoleuca melaleuca* (Pers.) Murrill had antimicrobial activities inhibiting Gram-negative *E. coli, Pseudomonas aeruginosa,* and *Acinetobacter baumannii*.

### 5.4. Filamentous Fungi

Yeasts mainly occur in milk, meat, food, and products such as fruit, yogurt, jams, sausage, and cheeses. Generally, antimicrobial compounds produced from yeasts inhibit the evolution/growth of pathogenic organisms (bacteria or molds) in food products. Some classes of yeasts secrete toxins, thereby naming them killer yeasts. Killer yeasts naturally occur in rotten vegetables and fruits and constrain the growth of other yeast strains and also inhibit microbial growth [167]. *Saccharomyces cerevisiae* (baker’s yeast), unicellular yeast, is the most widely studied microorganisms involved in many biotechnological practices because of its good fermentation capacity [168]. The inhibitory mechanism of *S. cerevisiae* killer strains was discovered in 1963 by Bevan and co-worker’s, and the phenomenon is related to the secretion of a protein toxin, k1, and k28 from the host that kills sensitive target pathogenic cells in a receptor-mediated approach without direct cell-to-cell contact [169]. Other genera producing killer toxins include *Cryptococcus, Candida, Kluyveromyces, Williopsis, Pichia, Debaromyces,* and *Zygosaccharomyces* [170]. The anti-bacterial capability of *S. cerevisiae* is attributed to:

(a) Secretion of inhibitory proteins,
(b) Production of extracellular protease,
(c) Stimulation of immunoglobulin A,
(d) Procurement and eradication of secreted toxins,
(e) Killer toxins, sulfur dioxide, etc.

Sequential re-pitching of *Saccharomyces* biomass is a common process during brewing. Therefore, yeast is reused many times before its final dumping [171]. Hence, yeast develops an adaptive response against oxidative stress like that of human cells, leading to the accumulation of vitamins (B6 and B12) and minerals (enzyme co-factors including zinc, manganese, and copper) in the yeast cell. Phenolic compounds are also adsorbed by *Saccharomyces* from the exterior medium, which increases the phenolic content and antioxidant activity within yeast cells [172]. Efficient means are required to disrupt yeast cell walls and separate the products of interest, which are further used for food applications. However, increasing consumer’ fears regarding the toxicity of killer yeast strains present in food and milk products constitutes a direct risk to public health. 

## 6. Microalgae

The antimicrobial activity of microalgae is due to the presence of phytochemicals, including indoles, acetogenins, terpenes, fatty acids, phenols, and volatile halogenated hydrocarbons (Table 7) [173]. Moreno et al. [174] reported that *Chaetoceros muelleri* extracts’ antimicrobial activity is due to their lipid configuration, whereas *Dunaliella salina’s* is attributed to the presence of *β*-cyclocitral, *α* and *β*-ionone, phytol, and neophytadiene. In natural environmental conditions, microalgal cells release fatty acids against predators and pathogenic bacteria. It is elucidated that these fatty acids act on bacterial cell membranes causing cell seepage, a decline in nutrient intake, and reduced cellular respiration, ultimately resulting in cell death [175]. 

Chlorellin, the first antibacterial compound from a microalga *Chlorella,* is composed of a mixture of fatty acid and was isolated by Pratt et al. [183]. Chlorellin was reported to inhibit the activity of both Gram-positive and Gram-negative bacteria. *Arthrospira platensis*, commercially known as *Spirulina* had MICs of 0.20% for *L. innocua* and *P. fluorescens,* and an MIC of 0.25% for *Serratia,* whereas minimal bactericidal concentration (MBC) value was 0.30% for all of these species [184]. HPTLC screening and GC–MS analyses were conducted to detect and screen the macroalgae’s antimicrobial compounds. Peptides, namely AQ-1756, AQ-1757, and AQ-1766 identified from *Tetraselmis suecica* exhibited an antibacterial activity resulting in decreasing cell viability (human embryonic kidney cells) (HEK293) up to 75% after 24 h of treatment. AQ-1766 was more active against Gram-positive than Gram-negative bacteria, with MBC values between 40 and 50 µM [185]. Mendiola et al. [186] demonstrated that lipid fractions obtained from *Chaetoceros muelleri* by the supercritical CO_2_ method have antibacterial activity against *Staphyloccocus aureus* and *E. coli*. In contrast, extraction via classic methods using hexane, dichloromethane, and methanol solvent did not result in any activity against *E. coli*. However, these studies were unable to elaborate the mode of action of these antibacterial compounds. 

Axenic microalgae co-culture can produce compounds with potent activity against pathogenic bacteria. Kokou et al. [187] reported that axenic cultures of *Tetraselmis chui, Chlorella minutissima*, *Isochrysis* sp. and *Nannochloropsis* sp. inhibit *Vibrio harveyi*. The potent activity of microalgal compounds against microorganisms requires further development in the search for drugs and food preservatives. Therefore, the exploitation in medicine deserves to be further investigated.

## 7. Discussion and Future Prospects

One of the significant challenges healthcare services face worldwide is the excessive use of antibiotics in medicine and food production, leading to microbiome disruption. With the outburst of antimicrobial resistance strains, there is a continuous decline in the antimicrobial drug pipeline, and it has become necessary to discover and develop new agents/metabolites to tackle antibiotic resistance. Novel compounds that target microbial resistance can be developed to regulate the huge risk posed by multi-drug resistance. However, the production cost needs to be reduced by isolating these compounds from natural sources such as microorganisms and then synthesizing them or modifying derivative compounds. Along with this, further research into their toxicity against human cells, their mode of action, in vivo effects, and their interactions with commonly available antibiotics must be conducted. After the discovery of penicillin, many drug discoveries from microbial sources were reported. In addition, the advancement of techniques such as genetic engineering during the 1970s opened the door to the ignored source, i.e., microbial metabolites [188]. 

Ample research is being conducted to search for novel antimicrobial agents from biological sources, including bacteria, actinomycetes, fungi, yeast, etc. Table 8 depicts selected commercially available antimicrobial products alongside their uses.

LAB producing bacteriocins are a promising candidate for the food industry as they help to extend shelf life and safeguarde consumers’ health. Actinomycetes, particularly *Streptomyces* spp., exhibited effective antagonistic activity and played a significant role in drug discovery and development. 

In the ongoing rearch for novel antibiotics, archaeocins have generally been overlooked, and further studies on purifications and characterizations of archaeocins and sulfolobicins are in progress, resulting in the economical production of bioactive compounds for pharmaceutical applications. It is desirable to expand our understanding of the effectiveness and use of other naturally occurring ribosomally-synthesized peptide antimicrobials to understand their implantation and survival strategies, and to quantitatively estimate their efficacy for future applications in the pharmaceutical and health care sectors. 

Fungi synthesized small quantities of bioactive compounds in response to explicit environmental conditions which cannot be reproduced easily in the laboratory. Therefore, to develop new antimicrobial drugs from these fungal metabolites, commercial-scale synthesis must be accomplished potentially through strain improvement, optimizing growth conditions, and incorporating techniques, such as metabolomics, genomics, and pathway engineering. Endophytic, filamentous, and marine-derived fungi also offer a suitable substitute against toxic, ineffective, and expensive antimicrobial drugs because they act as a warehouse filled with bioactive compounds with endless potential for biological properties. Antimicrobials, isolated from mushrooms, act as essential substitutes to synthetic drugs and preservatives, whose protection and influence on the health of humans, animals, and food are still uncertain. Although there are many edible mushrooms, the mushroom species identified have antimicrobial properties which are quite small. The current review demonstrates potent bioactive substances with antimicrobial activities from edible mushrooms. Hence, they must not be considered only as a culinary delicacy, but also taken as therapeutic agents. However, methods for isolation, purification, identification, and characterization of antimicrobial compounds from mushrooms need to be developed. 

Microalgae are a promising source of high-value products, and large-scale screening programs have been conducted to discover the antimicrobial potential of microalgal extracts against pathogenic and foodborne organisms. However, major antibacterial and antifungal activity reports were predominantly from the *Chlorella* sp. and *Chlamydomonas* sp. Many hurdles exist in developing the marine product, including resource supply issues, large-scale production, production cost, and determination of the efficacy target. These obstacles must be bypassed by optimizing mass culturing conditions, utilizing biotechnological techniques, etc. Along with these measures, extensive clinical trials will be needed to determine the in vivo fortune of antimicrobials from microbial extracts on mammalian cells. 

Therefore, developing and using robust screening and high-throughput methods will be essential to study their antimicrobial activity, thereby increasing the chances of discovering and identifying new antibiotic molecules. To achieve this goal, the experimental design must include all possible variables, such as recovering both intra- and extracellular extracts produced by microorganisms under variable growth conditions, utilizing potential inducers of antimicrobial activity, and testing these compounds against a more significant number of targets. In recent years, nanoencapsulation has gained much attention. It is a technique used for formulating and stacking a compound in nanosized carriers that can carry and deliver the molecules to the targeted site. Nanoencapsulation allows the conservation and controlled release of bioactive compounds, followed by resistance to pH and temperature variations, lesser product contamination, economic viability, and stability. Chromatographic separation techniques were used recently for purifying antimicrobials, followed by their chemical characterization using spectroscopic techniques, and response surface methodology (RSM) to predict the yield of the crude antimicrobial extract. Detailed functional and structural knowledge would explain antimicrobials’ mode of action and performance at cellular and molecular levels. However, for this, a better understanding of the structure, function and, existing mode of action of newly identified antimicrobials is required.

## 8. Conclusions

In conclusion, microorganisms are probable sources of bioactive compounds, and this review has explored of microorganisms’ aptitudes to deliver novel bioactive compounds with potential pharmaceutical and nutraceutical applications. Microorganisms have fascinated many researchers due to the ease of growth and understanding of their chemical interactions. The development of new biotechnological tools and techniques contributes to the discovery of next-generation antimicrobial compounds. The application of microorganisms in human foods, animal feeds, agriculture, and an increased market demand motivates the research and development of novel antibiotics and preservatives. Furthermore, the molecular docking and structural analysis approaches can design potent pathogen-specific antimicrobial agents that exhibit lesser toxicity, higher selectivity, and biodegradability. Therefore, exploiting microbial biodiversity and biotechnological potential to discover novel bioactive compounds to treat life-threatening diseases and safeguard human health.

## Figures and Tables

**Table 5:** Antimicrobial compounds extracted from endophytic fungi.

Compound	Chemical Structure	Producer	Active Against	Host	References
e 1, 4-naphthoquinone derivatives	*-*	*Talaromyces* sp. *SK-S009*	*Pseudomonas* sp.	*Kandelia obovata*	[128]
Clavatol	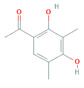	*Aspergillus clavatonanicus,* *Aspergillus elegans KUFA0015*	*Botrytiscinerea, Didymella bryoniae, Fusarium oxysporum f.* sp. *cucumerinum, Rhizoctonia solani,* and *Pythium ultimum*	*Taxus mairei,* *Monanchora unguiculate* (Marine sponge)	[129]
Lactones	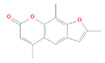	*Phomopsis* sp. YM 311483	*A. niger, Botrytis cinere,* and *Fusarium*	*Azadirachta indica*	[130]
Jesterone	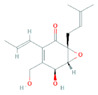	*Pestalotiopsis jesteri*	*Pythium ultimum, Phytophthora citrophthora, Rhizoctonia solani* and *Sclerotinia sclerotiorum*	*Fragraea bodenii*	[131]
Peniciadametizine A	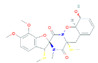	*Penicillium adametzioides* AS-53, *Penicillium janthinellum strain HDN13-309*	*Alternaria brassica*	Sponge collected at the Hainan Island of China, roots of *Sonneratia caseolaris*	[132]

**Table 6:** Antimicrobial compounds extracted from marine fungi.

Compounds	Structure	Producer	Active Against	Environment Source	References
Penicisteroid A	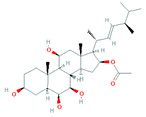	*Penicillium chrysogenum* QEN-24S	*A. niger* and *Alternaria brassicae*	Marine algae associated *Penicillium* sp.	[135]
Arisugacin K	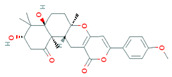	*P. echinulatum*	*E. coli*	Marine alga *Chondrus ocellatus.*	[136]
Methyl (Z)-3-(3, 4-dihydroxyphenyl)-2- Formamidoacrylate	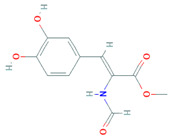	*P. oxalicum* EN- 290	*S. aureus*	Marine algae associated *Penicillium*	[137]
Chermesins A and B	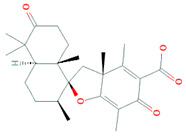	*P. chermesinum* EN-480,	*C. albicans,* *E. coli, M. luteus,* and *V. alginolyticus*	Marine algae associated *Penicillium*	[138]
Comazaphilones C (C–E)	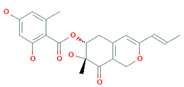	*P. commune* QSD-17	*Antibacterial*	*Penicillium* sp. from marine sediments	[139]
Penicibilaenes A	*-*	*P. bilaiae* MA-267	*Colletotrichum gloeosporioides*	Rhizospheric soil of *Lumnitzera racemosa*	[140]
Xylarinonericin D and E	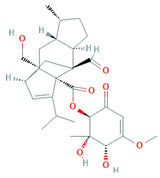	*Penicillium* sp. H1	*Fusarium oxysporum f.* sp. *Cubense* (antifungal)	Beibu Gulf nearby Guangxi	[136]
Terretonin G	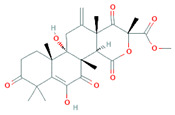	*Aspergillus* sp. *OPMF00272*	*S. aureus* FDA209P*, Bacillus* *subtillis* PCI219 and *Micrococus* *luteus* (ATCC9341)	Ishigaki island	[141]
Schevalone E	*-*	*A. similanensis* sp. *nov.*	MRSA	Sponge *Rhabdermia* sp. from the coral reef of the Similan Island	[142]
Asperitaconic acids A–C	*-*	*A. niger* LS11	*S. aureus*	Sponges-associated *Aspergillus* sp.	[143]
Ochramide B	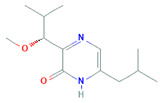	*A. ochraceus* LCJ11-102	*Enterobacter* *aerogenes*	Marine sponge *Dichotella gemmacea*	[144]
Spiculisporic acids F and G	*-*	*A. candidus* HDf2	*P. solanacearum* and *S. aureus*	Marine animals associated *Aspergillus* sp.	[145]
Aspergicin	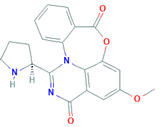	*Aspergillus* sp. FSY-01 and *Aspergillus* sp. FSW-02	*S. aureus, S. epidermidis, B. subtilis, B. dysenteriae, B.* *proteus* and *E. coli,*	Mangrove *Avicennia marina* in Guangdong.	[146]
Asperamide	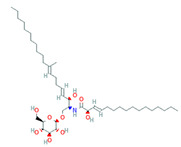	*A. niger* EN-13	*C. albicans*	Marine algae associated *Aspergillus* sp.	[147]
Flavusides A and B	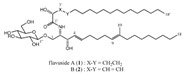	*A. flavus*	*S. aureus* and MRSA	Marine algae associated *Aspergillus* sp.	[148]
Isorhodoptilometrin- 1-methyl ether	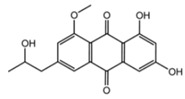	*A. versicolor*	*B. subtilis, B. cereus* and *S. aureus*	Marine algae associated *Aspergillus* sp.	[149]
Asperterrein	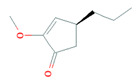	*Paecilomyces lilacinus* EN-531 and *A. terreus* EN-539	*Alternaria brassicae, E. coli, Edwardsiella* *tarda, Physalospora piricola,* and *S. aureus*	Marine algae associated *Aspergillus* sp.	[150]
Speradine A	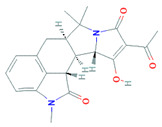	*A. tamarii* M143	*Mycrococcus luteus*	driftwood in Okinawa	[136]
Versiperol A	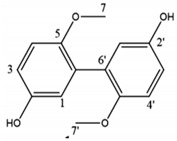	*A. versicolor* MCCC 3A00080	*S. aureus*	seawater-associated *Aspergillus* sp.	[151]
Ergosterdiacids A and B	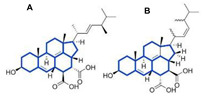	*Aspergillus* sp.	*M. tuberculosis*	Marine sediments associated *Aspergillus* sp.	[152]
Heptapeptide RHM1	*-*	*Acremonium* sp. HM1	*S. epidermidis*	Marine sponges- associated fungi	
Trichoderins A	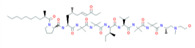	*Trichoderma* sp. 05FI48	*M. smegmatis*	Marine sponges- associated fungi	[153]
Botryorhodines I and J	*-*	*Setosphaeria* sp. SCSIO 41009	*Colletotrichum* *asianum*	Marine sponge *Callyspongia* sp.	[154]

**Table 7:** Selected antimicrobial extracts from microalgae.

Microalgae	Target Microorganism	Active Extract	References
*Scenedesmus quadricauda*	*S. aureus* and *P. aeruginosa*	Methanolic extract	[176]
*Tetraselmis* sp.	*E. coli*, *P. aeruginosa*, and *S. aureus*	Ethanolic extract	[177]
*Phaeodactylum tricornutum*	*Listonella anguillarum*, *Lactococcus garvieae*, *Vibrio* spp. and MRSA	Eicosapentaenoic acid	[175]
*C. vulgaris*	*Steinernema feltiae*	Hydrophilic extracts	[178]
*Skeletonema costatum*	*Listeria monocytogenes*	Extra-metabolites	
*S. costatum*	*Vibrio* spp., *Pseudomonas* sp. and *Listeria monocytogenes*	Unsaturated, saturated long-chain fatty acids	[179]
*Haematococcus pluvialis*	*E. coli*, *S. aureus*, *Candida albicans*	Short-chain fatty acids (butanoic acid and methyl lactate), Astaxanthin	[180]
*Amphidinium* sp.	*A. niger*, *Trichomonas foetus*	Karatungiols	[181]
*Chlamydomonas reinhardtii*	*A. niger*, *A. fumigatus*, *C. albicans*, *S. aureus* and *E. coli*	Methanolic extracts	[182]

**Table 8:** List of selected antimicrobial compounds with commercial trade name and uses.

Compounds	Brand Name	Company Name	Country	Uses	References
Nisin	Nisaplin^®^	Danisco	Denmark	Used as a food preservative	[189]
Nisin	Novasin^™^	Danisco	Denmark	Used as a food preservative	[189]
Nisin	Delvo^®^Nis	DSM	Netherlands	Used as a food preservative	[190]
Nisin	Chrisin^®^	Chris Hansen	Denmark	Used as a food preservative	[190]
Nisin	-	Duke Thomson’s International	India	Used as a food preservative	[191]
Nisin	-	Ecobio Biotech Co. Ltd.	China	Used as a food preservative	[191]
Delvocid-Natamycin	-	Duke Thomson’s International	India	Used as a food preservative	[191]
Natamycin	Delvocid^™^	DSM	Netherlands	Used as a food preservative	[191]
Daptomycin	Cubicin	Novartis India Ltd.	India	Used to treat bacterial infections	[192]
Lipopeptides	RhizoVital^®^	ABiTEP, GmbH	Germany	Biological control in agriculture	[191]
Lipopeptides	Kodiak^™^	Gustafson Inc.	USA	Biological control in agriculture	[191]
Lipopeptides	Taegro^®^	Novozymes	USA	Biological control in agriculture	[191]
Lipopeptides	Serenade^®^	AgraQuest Inc.	USA	Biological control in agriculture	[191]
Lipopeptides	Botrybel	Agricaldes	Spain	Biological control in agriculture	[193]
Spironolactone	Aldactone^®^	Pfizer Medical	USA	To treat various diseases	[194]

## Data Availability

Not applicable.
